# IMPLEMENTING THE AGE-FRIENDLY HEALTH SYSTEM INTO CVSMINUTE CLINICS

**DOI:** 10.1093/geroni/igz038.538

**Published:** 2019-11-08

**Authors:** Mary A Dolansky, Anne pohnert

**Affiliations:** 1 Case Western Reserve University, Cleveland, Ohio, United States; 2 CVSMinute Clinic, Rhode Island, United States

## Abstract

Each day, 10,000 individuals in the United States turn 65 and nearly 30% of our nation’s 884 million ambulatory care visits involve patients ages 65 and older. With this trend, the use of convenient care settings for treatment for chronic conditions associated with aging such as diabetes and hypertension is emerging. The need for the delivery of age-friendly care in convenient care settings is essential to ensure high quality and safe care. CVS MinuteClinic® and the Institute for Healthcare Improvement (IHI) will present the plan and preliminary findings for the extension of the IHI’s Age-Friendly Health Systems initiative into the CVS MinuteClinic. The Age-Friendly Health System model includes 4 M’s: What Matters, Medications, Mentation, and Mobility as essential components for care delivery. The presentation will include (1) the partnership with the IHI’s Age-Friendly Health System Action Community, (2) a demonstration of the Age-friendly Virtual Clinic and learning platform that includes training modules and practice-based tools to facilitate assessment and action steps for the implementation of the 4Ms based on Age-Friendly Health System content, and (3) the evaluation logic model that will be implemented. The implementation plan will provide the framework to embed age-friendly process and outcome measures into the CVS MinuteClinic electronic health record platform, Epic. The project will impact all 1,100 CVS MinuteClinic locations across the nation.

